# The (Neuro)-Science Behind Resilience: A Focus on Stress and Reward

**DOI:** 10.32872/cpe.11567

**Published:** 2023-03-31

**Authors:** Chantal Martin-Soelch

**Affiliations:** 1 IReach Lab, Unit of Clinical and Health Psychology, Department of Psychology, University Fribourg, Fribourg, Switzerland

Mental disorders represent one of the major causes of disability worldwide, with depressive disorders being the leading causes of burden among mental disorders in all age categories above 14 years old, followed by anxiety disorders (GBD 2019 Mental Disorders Collaborators, 2022). Although knowledge concerning their etiology has improved, it is still unclear why one person will develop a mental disorder while another will not when facing adversities. In this context, the identification of resilience mechanisms is crucial. Adopting an approach based on mechanisms allows us to have a transdiagnostic and transtheoretical approach and to target specific processes for the development of psychological interventions.

Indeed, better knowledge of resilience mechanisms allows the development of targeted interventions in at-risk populations. Some risk factors for the development of mental disorders have been well identified, such as childhood abuse or in general early adverse childhood experiences (ELS) (Kessler et al., 2010; Mandelli et al., 2015). ELS have received increased attention from research, which led recently to the development of a consortium in the framework of the global traumatic stress collaboration dedicated to the investigation of socio-emotional consequences of ELS (Pfaltz et al., 2022). A further well-investigated risk factor is being the offspring of one or more parents suffering a mental health condition, particularly depression, bipolar disorder or schizophrenia (Rasic et al., 2014).

Resilience can be defined as the capacity of an individual to adapt successfully to highly adverse events and keep a healthy functioning by harnessing resources (Southwick et al., 2014). It is most often measured by questionnaires, although these may be limited by issues of internal validity in particular because little is known about the different elements that make up resilience. These questionnaires therefore often focus on one or a set of measures related to well-known protective factors or resources, such as feelings of self-efficacy, self-esteem, sense of mastery, optimism, positive affect, good emotion regulation skills or sense of coherence (Southwick et al., 2014). Neuroscience has provided new insights in this area and indicates that neurocognitive and neuroaffective factors, such as cognitive flexibility or reactivity to stress or reward may play a role in resilience. These two processes- reward and stress- are linked to well-defined brain systems that are considered to be crucial for human motivation and adaptation (Godoy et al., 2018; Schultz, 2000). Blunted neural responses to reward have been consistently observed in depressive disorders, and have been hypothesized to underly the symptoms of anhedonia, apathy and loss of interest observed in these conditions (Pizzagalli et al., 2009). And a large body of empirical evidence shows the importance of stress in the development of several psychopathological conditions, among others depression (Liu & Alloy, 2010). Recently, it has been postulated that not only the responses to reward or the effect of stress, but rather an interaction between both is involved in the etiology of mental disorders. Thus, a high reactivity of the brain to stress and a reduced brain reactivity to reward, also conceptualized as an imbalance between the neural responses to stress and to reward, has been hypothesized to be a vulnerability factor for the development of mental disorders, in particular depressive disorders (Admon et al., 2013). This model has been completed with research works showing that not only the neural responses during the presentation of stressful stimuli or rewarding information is important, but also the neural recovery after these events, in particular longer recovery after stress and shorter recovery after reward, might play a role. This has been conceptualized as emotional inertia and brought in relationship with difficulties in emotion regulation (Koval et al., 2015).

Our laboratory, the IReach lab at the Department of Psychology of the University of Fribourg (Switzerland), has been particularly interested in the stress-reward interactions and their role in understanding the development of disorders in a transdiagnostic approach based on clinical neuroscience research results. Preliminary studies from our group suggest that in children of parents suffering from depression, reactions to rewards are impacted differently than in a control group under acute stress conditions (Gaillard et al., 2020; Martin-Soelch et al., 2020). These results are interesting because our participants had no clinical symptoms, but they showed different neural activation to reward stimuli and to the effect of stress on their processing. This may suggest a form of latent vulnerability that is not observable at the behavioral level. These results are in line with differences observed in response to rewarding information (without stress) in offspring of depressed parents (McCabe et al., 2012).

**Figure 1:**
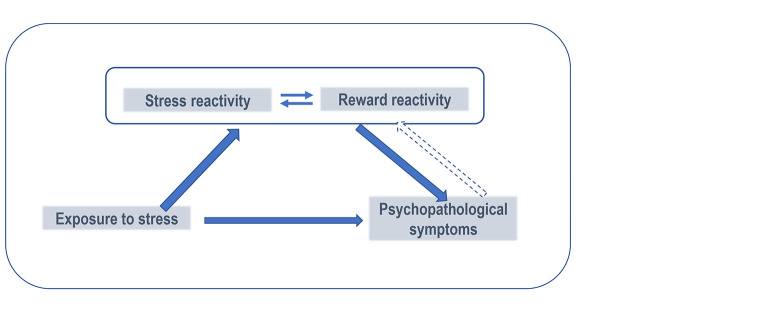
Simplified Schematic Model of the Stress-Reward Interaction as Mediator of the Relationship Between Stress Exposure and the Development of Psychopathological Symptoms *Note.* A higher neural reactivity to stress and a lower reactivity to reward are hypothesized to be a vulnerability factor for psychopathology. Interventions targeting one or both mechanisms can be used in a preventive manner or as treatment.

Understanding and integrating the interactions between the reward and stress systems in a model (see Figure 1) can serve as basis for developing and testing psychological prevention and/or treatment interventions that target these mechanisms. On this basis, we developed for instance a multi-modal stress management program that has shown effects in activating resources in general and increasing the feeling of reward in daily life in particular (Recabarren et al., 2019). Other therapeutic programs have also shown significant effects on reward processing. For instance, a study by Dichter et al. (Dichter et al., 2009) suggests that behavioral activation restores the brain's reactivity to reward in association with improvement of depressive symptoms in individuals diagnosed with major depressive disorder. Furthermore, a recently developed and validated intervention, the Mindfulness-Oriented Recovery Enhancement (MORE) program, which was originally developed for the management of substance use and addiction problems, in particular opioid use in connection with chronic pain management (Garland et al., 2022), also seems to show beneficial effects on the brain's responses to reward and to increase positive affect and emotion regulation (Garland et al., 2017). This group intervention program combines cognitive-behavioral techniques, mindfulness and meditation methods with savoring training. This 8-weeks training has shown significant beneficial effects on opioid use and improvement in chronic pain symptoms in large clinical trials in the USA (Garland et al., 2022). A current study of our group is interested in investigating whether this program can improve pain symptoms as well as affective symptoms of women suffering from fibromyalgia at a clinical level and to investigate as well the neural responses to reward changes and in the functioning of dopamine, a neurotransmitter that has been linked to reward, before and after the intervention (Ledermann et al., 2021).

**What Does This Mean for Psychologists?**

Integrating results and approaches from other disciplines such as neuroscience to understand and identify neural mechanisms that are important for the development of disorders and that are directly associated with psychological mechanisms allows the development of targeted psychological interventions that can be used preventively in groups of individuals at risk of developing psychological disorders, for example in offspring of depressed parents. These interventions can also be used in addition to or in complement to usual psychotherapeutic treatment for individuals currently diagnosed with a mental disorder in order to offer targeted treatment. These mechanism-based interventions enrich the clinical psychologist's range of interventions and allows for a transdiagnostic approach. Finally, as their neural correlates are known, it is possible to perform neuroimaging measures of these mechanisms before and after the intervention in randomized controlled trials to show the effect of the psychological interventions not only at a clinical level, but also at a neural level. This approach is therefore a promising avenue for the development of new clinical psychological interventions either for the prevention or the treatment of mental disorders.
